# Imaging Oxygen Concentrations in Bone Scaffolds during Cellular Activity and Fluid Perfusion

**DOI:** 10.1021/acsbiomaterials.4c01845

**Published:** 2025-06-04

**Authors:** Hannu Välimäki, Karim Ameziane, Sriparna Bhattacharya, Jonathan Massera, Pasi Kallio, Jeffrey N. Anker

**Affiliations:** † Micro- and Nanosystems Research Group, Faculty of Medicine and Health Technology, 7840 Tampere University, Tampere 33014, Finland; ‡ 3Bs: bioceramics, bioglasses and biocomposites, Faculty of Medicine and Health Technology, Tampere University, Tampere 33014, Finland; § Department of Physics and Astronomy, 2545 Clemson University, Clemson, South Carolina 29634, United States; ∥ Departments of Chemistry and Bioengineering, Clemson University, Clemson, South Carolina 29634, United States

**Keywords:** Bone scaffolds, bioglass, oxygen imaging, perfusion, luminescence

## Abstract

Developing bone replacement scaffolds has been a driving ambition of regenerative medicine. Although great progress has been achieved for small scaffolds, the real clinical need is for large scaffolds >5 mm. Oxygenating these scaffolds is challenging, as slow diffusion rates lead to necrotic regions in the scaffold core. In this work, we modulate *in vitro* oxygen concentration in a scaffold in a flow chamber using an external perfusion pump while imaging oxygen concentrations below the scaffolds. With no external flow, yeast cells growing in the scaffold deplete oxygen, especially from the center, with concentrations reaching a steady state consistent with reaction-diffusion models. The oxygen is restored via pumping fresh medium through the scaffold. The oxygen profiles are highly reproducible from cycle to cycle. This lays the groundwork for future *in vivo* oxygen imaging studies using localized light sources and external perfusion pumps for modulation.

## Graphical abstract

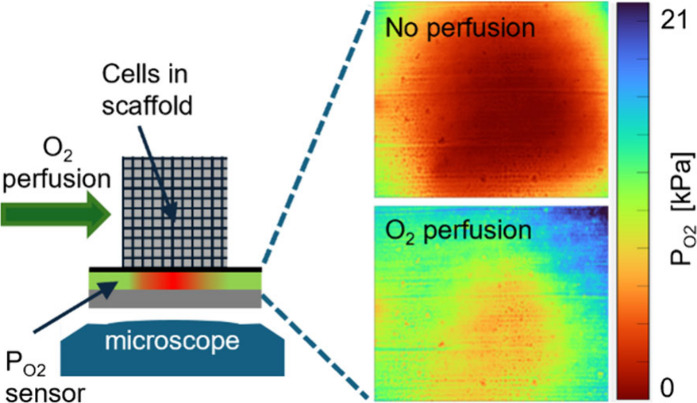

## Introduction

Patient quality of life is profoundly impacted by large bone defects, which are usually caused by bone cancer or high-energy traumatic injuries. For example, extremity bones affect patient mobility and ability, while facial and mandible bones affect appearance, facial expressions, vocalization with tongue motion, and chewing/swallowing. These injuries are typically treated by harvesting bone from a secondary site, but this unfortunately necessitates prolonged surgery, risks secondary site morbidity, and is not always successful.^1^

Developing bone replacement scaffolds has been a driving ambition of regenerative medicine.^2^ Much progress has been achieved, especially in *in vitro* and in small animal models. However, the real clinical need is for large scaffolds, which suffer from three problems related to oxygenation and cell growth:^3–5^ (1) since vascularization has been difficult to generate and connect to the blood supply,^6^ oxygen and nutrient diffusion and transport into the scaffold core is insufficient for cell growth or to remove waste, leading to necrotic regions; (2) the large surface area of the implanted scaffold, coupled with often open wounds and necrotic regions in the scaffold with insufficient vascularization, makes infection an acute risk with devastating consequences (antibiotics are generally ineffective once biofilms are established and implant removal is usually required); (3) the large scaffold size leads to significant torque on the implanted scaffold and fixation hardware, and slow bone healing from poor oxygenation gives time for mechanical fatigue and failure.

Researchers have proposed many approaches to enhance tissue oxygenation and cell growth, ranging from promoting angiogenesis^7^ to oxygen releasing scaffolds,^6,8^ to adding photosynthetic algae and exciting with light,^9^ to hyperbaric chambers.^10^ In addition, ultrasound is clinically used to stimulate bone growth^11^ and potential mechanisms include increased perfusion, heating, and mechanical stimulation. However, it is unclear how any of these approaches actually affect oxygenation *in vivo* and how this correlates with bone growth (as well as potential pathologies from either hypoxia or hyperoxia). Studies often use micro-CT and post-mortem histology to examine bone growth, but measuring oxygen through bone and tissue at submillimeter resolution is challenging unless electrodes or optodes are used to puncture the tissue and bone, which would locally injure the tissue and potentially create a physical channel effecting its oxygenation.

The Kallio group (co-authors) has developed and applied luminescence lifetime based^12,13^ and ratiometric methods to measure and image oxygen concentration in microfluidic chips while modulating the oxygen flow and gradient with microfluidics. The methods have been useful for hypoxic studies with cardiomyocytes^14,15^ and hepatocytes,^16^ as well as for characterization of various chips with oxygen modulation possibility.^17^ However, the methods have not previously been applied to oxygen imaging in thick samples such as bone scaffolds. Our model here is *in vitro*, with a scaffold placed on a luminescent oxygen-sensing film on a glass slide. In future, we plan to extend this to X-ray excited optical luminescent films which the Anker group developed for high resolution imaging through soft tissue in human cadaveric models^18–20^ and up to 2 cm of bone and tissue in live rabbit models.^21,22^ Early work has also shown feasibility for spectrochemical X-ray luminescence oxygen measurements *in vitro*.^23^ We are particularly interested in modulating oxygenation in bone scaffolds via external perfusion. The external perfusion is both a convenient way to reproducibly modulate oxygenation and test the oxygen imaging. It is also a potentially viable clinical approach, although to date only used for continuous localized antibiotic therapy of infections of native bone with metal implants.^24,25^ In summary, this letter describes an important step in our longer term goal of modulating oxygen and other drugs in large bone scaffolds using external perfusion pumps, and quantifying how these affect local oxygenation, bone growth, and infection. Towards this goal, we show the first oxygenation imaging beneath a bone scaffold during cell growth and perfusion *in vitro*.

## Materials and Methods

### Scaffold Fabrication

The ink was prepared using Pluronic F127 (30 wt % in DI water) and glass powder (1393B20, particle size <38 μm).^26^ The pluronic/glass ratio was 30:70 wt %. The ink was homogenized using a Vibrofix VF1 electrical shaker (IKA-Labortechnic, Staufen, Germany) at 2500 rpm. The ink was then vortexed with at least five mixing-cooling cycles (30 s mixing + 30 s cooling in an ice bath) until the ink was homogeneous and did not show any visible bubbles. Finally, the ink was loaded into an Optimum 3 cm^3^ printing cartridge (Nordson EFD, Bedfordshire, England) and left stabilizing for 20 min at room temperature.

A 3Dn-Tabletop printer (nScrypt Inc., Orlando, FL, USA) was used for robocasting 3D porous scaffolds. The cartridge was attached to the 3D printer, and the ink was extruded through the SmoothFlow Tapered Tips with a tip diameter of 0.41 mm (Nordson EFD Optimum SmoothFlow, Westlake, Ohio, USA). The ink was extruded onto an acrylic sheet (Folex AG, Seewen, Switzerland). The material feed was set to ∼15.0–25.0 psi to maintain a continuous flow during movement of the tip. After being dried at room temperature for at least 24 h, scaffolds were sintered to allow fusing of glass particles and to remove the binder. The robocasted scaffolds had bottom dimensions of 6.5 mm × 6.5 mm and height of 5.5 mm (Figure 1A). The scaffolds had an average porosity of 43% and pore size of ∼200 μm, as shown in the SEM image (Figure 1B).

**Figure 1:**
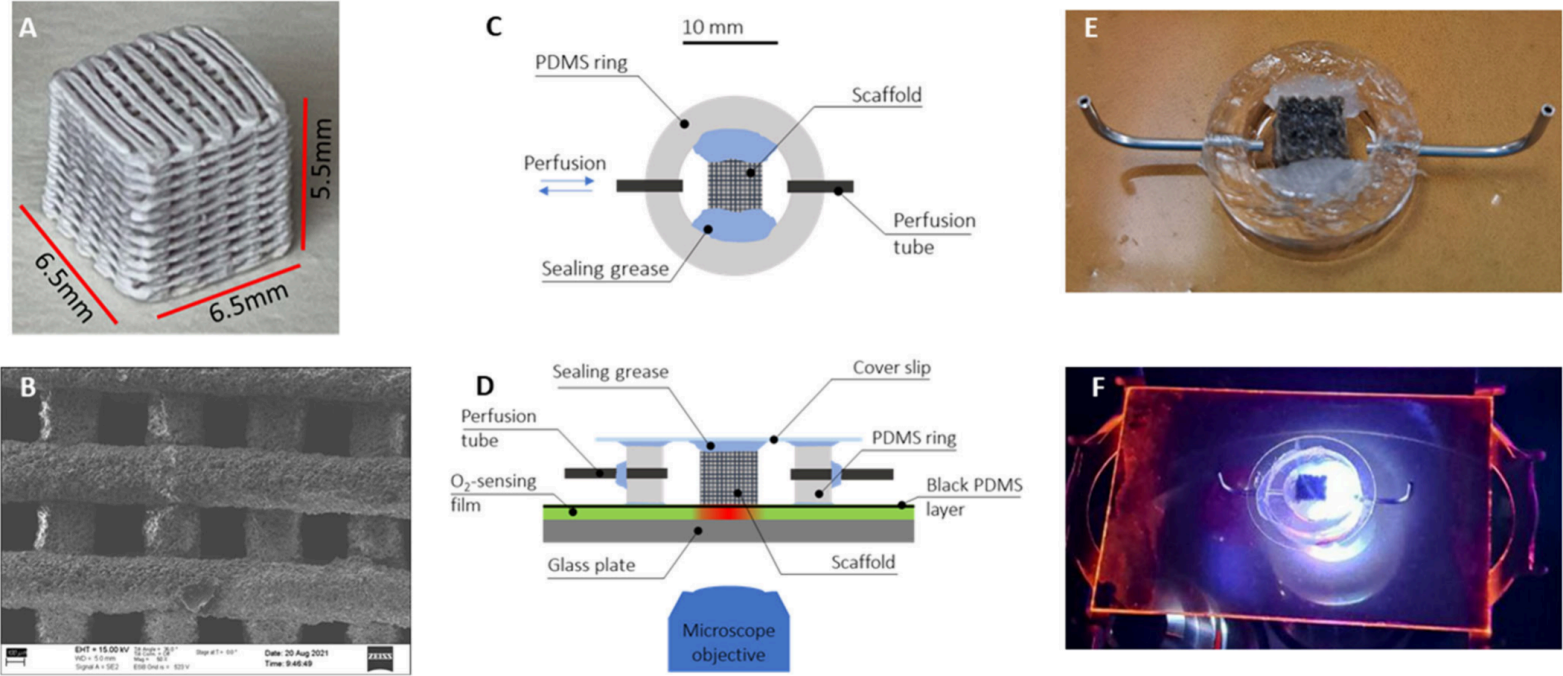
(A) 3D printing of bioactive glass scaffold. (B) SEM picture of the porous scaffold. (C) Schematic representation of the perfusion chamber seen from above without a coverslip and sensor plate. (D) Schematic cross-section of the device mounted onto the oxygen sensor plate and sealed with a coverslip. (E) Picture of the device mounted onto the sensor plate before sealing with a coverslip. (F) Whole device mounted into the microscope.

### Yeast Incubation

Approximately 1 g of baker’s yeast was suspended into 5 mL of aqueous 3 wt % glucose solution. The scaffold was then soaked in the yeast-containing solution for 10 min. After soaking, the scaffold was placed into a Petri dish, and a few drops of glucose solution were added onto it. The yeast-containing scaffold was then left to incubate in the Petri dish at room temperature for 10 min.

### Oxygen Imaging

The ratiometric oxygen imaging method according to Ungerböck et al.^27^ was applied in a widefield fluorescence microscope (Zeiss Axio Observer Z1) equipped with a color camera (Axiocam 503), collimated LED source (M455L4-C4, 455 nm, 690 mW, Thorlabs), and FITC-filter set, consisting of a bandpass 450–490 nm excitation filter, a 510 nm dichroic mirror, and a long-pass 515 nm emission filter. Briefly, the red channel of the camera acts as the oxygen-sensitive channel, while the green channel acts as the reference. Platinum­(II) 5,10,15,20-tetrakis­(2,3,4,5,6-pentafuorphenyl)­porphyrin (PtTFPP) (LivchemLogistics GmbH, Frankfurt, Germany) was used as the oxygen-sensitive indicator and Macrolex Fluorescent Yellow (MFY) (Livchem Logistics GmbH, Frankfurt, Germany) as the reference dye.

### Oxygen Sensing Films

Polystyrene (PS) pellets (Sigma-Aldrich), PtTFPP, and MFY were dissolved in CHCl_3_, and oxygen sensing films were fabricated on glass plates (50 mm × 75 mm × 1 mm, Gerhard Menzel GmbH, Braunschweig, Germany) by knife coating, as described in detail by Tornberg et al.^17^ The oxygen sensing films had an approximate thickness of 14 μm. The films were then treated with oxygen plasma (Pico, Diener Electronic GmbH+Co. KG, Ebhausen, Germany) for 20 s, and a black PDMS layer (approximate thickness of 20 μm) was spin-coated on top of the sensing film. The black PDMS film was manufactured by adding black pigment (Bone Black P.B.K:9, Kymin Palokärki, Finland) in a ratio of 1:2 with respect to the curing agent of the PDMS. The PDMS film facilitates the bonding between the PDMS well and the oxygen sensing film, and the black pigment improves the robustness of the oxygen imaging system against many optical disturbances,^28^ including ambient light and especially the reflections from the mounted scaffold grid. A 20 μm PDMS film adds an extra diffusion layer on top of the oxygen sensing film, thus increasing the sensor response time. However, our previous studies show that the arrangement can still be used for recording oxygen concentration modulations with time constants under 20 s,^17^ which is more than enough for the present study.

### Perfusion Chamber

Figure 1C shows a schematic representation of the perfusion chamber seen from above without a coverslip and the sensor plate. The chamber was created by cutting a 6.0 mm thick ring of PDMS using punches 19 and 11 mm in diameter for the outer and inner walls of the ring, respectively. Holes (0.8 mm) for the perfusion inlet and outlet were punched at the opposing sides of the ring. Metal connector tubes were subsequently inserted into the inlet and outlet holes and sealed with grease to provide the perfusion tubes.

Figure 1D shows a cross-section of the device when mounted onto the oxygen sensor plate and sealed with a coverslip. Sealing grease was added to the opposing inner sides of the chamber, as shown in Figure 1E, to secure the scaffold and seal its sides. The scaffold was then inserted to the center of the chamber. The greased coverslip was used to seal the chamber from above. Finally, the scaffold had two open faces perpendicular to the flow direction. This arrangement allowed perfusion only through the scaffold structure, as opposed to around it. Figure 1F shows the whole setup when mounted on the microscope.

We note that the perfusion chamber does not allow for vertical flow or diffusion through the glass substrate walls of the chamber. When there is no flow (i.e., after the flushing step) and diffusion dominates, the (zero) vertical oxygen transport through the wall is, according to image theory, essentially equivalent to the (zero) vertical net transport over the centerline of a scaffold twice as thick (diffusion down and up are equal by symmetry).

### Experimental Arrangements

The assembled device on the oxygen sensing plate was placed into the microscope, and silicone tubes were connected to the inlet and outlet of the device. The inlet and outlet silicone tubes were connected to a 10 mL syringe containing the glucose solution and a waste container, respectively. The perfusion chamber was carefully filled with the glucose solution before the measurement. The measurement was carried out in three successive cycles. The first cycle was 31 min long with a sampling interval of 1 min. The second and third cycles were measured with an increased sampling rate of 30 s and a prolonged duration of 51 min. For all measurements, manual back-and-forth flushing was applied from the inlet. The back-and-forth flushing flow rate was estimated to be between 3 and 5 mL/min. In each cycle, flushing started after 5 min of baseline and continued for 3 min.

### Data Analysis

The luminescence color images (300 ms exposure time, 30–60 s sample interval) were processed using MATLAB. Each color image (1460 × 1936 pixels) was transformed into a ratiometric image by dividing the intensity of the red channel pixelwise by the green. To reduce both the noise (originating mostly from the imperfections of the sensing film) and processing time, the size of the ratiometric image was reduced to 145 × 193 pixels by areal averaging (digital binning). This smoothed the oxygen values and provided an acceptable effective pixel size of ∼36 μm (∼0.6% of the scaffold dimension of 6.5 mm). The ratiometric image was transformed into an oxygen image by applying the modified two-site Stern–Volmer model^29^ with the same parameter values for the whole image. The model parameters were estimated by (i) recording the average ratiometric responses at *P*_O2_ = 0.0 kPa and *P*_O2_ = 20.2 kPa in the experimental setup and (ii) using *f*_1_ = 0.712 for the quenchable fraction of the indicators, determined in the separate calibration setup (see Figure S2 in the Supporting Information). The calibration method does not take into account possible inhomogeneities in the oxygen sensing film. Finally, the measured oxygen tension (or oxygen partial pressure) *P*_O2_ [kPa] and the corresponding dissolved oxygen concentration *C* [mol/L] are linked to each other through Henry’s law *C*(*x*,*t*) = *H* ^CP^ *P*_O2_(*x*,*t*), where *H* ^CP^ [mmol L^–1^ Pa^–1^] is the Henry solubility constant.^30^ In the remainder of the paper, we do the analysis mostly in terms of oxygen tension, but the reader may easily convert the tension values into dissolved concentration values using a typical solubility constant for oxygen in water at laboratory conditions:^30^ *H* ^CP^ ≈ 1.3 × 10^–5^ mmol L^–1^ Pa^–1^.

## Results and Discussion

Figure 2 shows the time-stamped oxygen tension images of the scaffold bottom during three consecutive measurement cycles. Although we observe a small degree of reflection from the scaffold in the raw red and green channel images (Figure S1a,b), this is a minor effect because the black PDMS layer attenuates reflections, and the ratio between red and green channels accounts for most of the common mode noise (Figure S1c). Instead, the primary feature is from oxygen modulation, which occurs beneath the scaffold. There are also some minor but visible artifacts originating from imperfect oxygen sensing film preparation, especially horizontal streaks from the knife coating procedure used to apply the film to the glass substrate. However, averaging over several pixels smooths these periodic streaks out.

**Figure 2:**
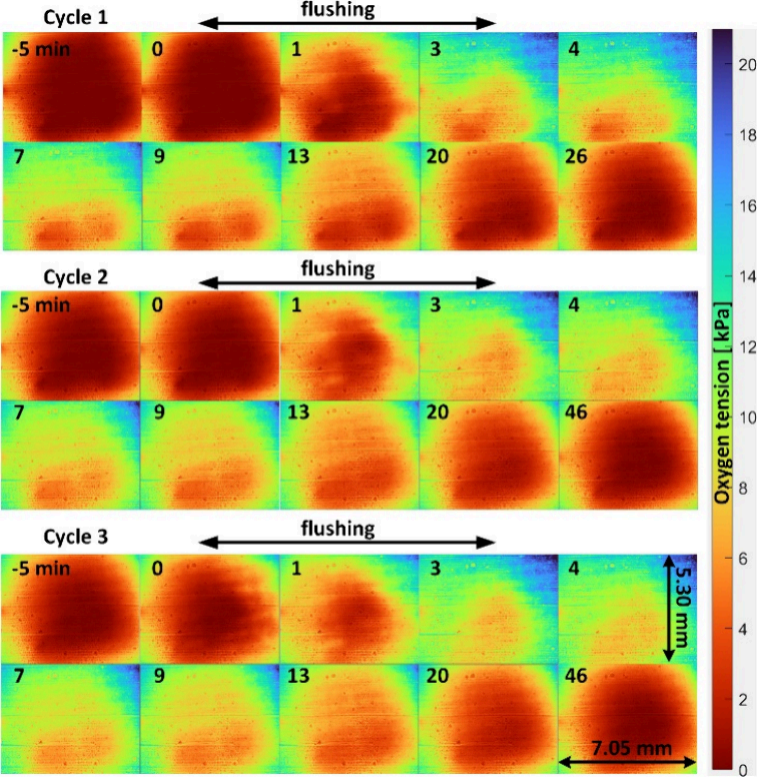
Time-stamped images of oxygen tension beneath the scaffold during three consecutive measurement cycles. The scaffold contains yeast cells which metabolize oxygen. In each cycle, the scaffold is flushed with fresh medium for 3 min between the time stamps *t* = 0 and *t* = 3 min and then let to stabilize for 23 min (Cycle 1) or 43 min (Cycles 2 and 3).

In each cycle, the time stamp *t* = 0 min denotes the start of the fluid flushing step. During the 5 min period before the flushing step (*t* ≤ 0 min), the oxygen images reveal highly depleted oxygen conditions beneath the scaffold, which are relatively stable in time. Flushing (in horizontal direction) starts at *t* = 0 and ends at *t* = 3 min, providing fresh medium and oxygen to the cells. This results in a sequential increase in oxygenation. After the flushing ends (*t* > 3 min), the hypoxic conditions inside the scaffold gradually return. This is repeated three times, and each cycle has similar images in time, showing high reproducibility. Interestingly, the lower part of the image seems to remain more hypoxic during all three cycles. This repeating asymmetry could be due to uneven flushing efficiency or inhomogeneous cell distribution in the scaffold. The first measurement cycle ended after 31 min (t = 26 min) while the second and third cycles ended after 51 min (t = 46 min) to ensure stationary conditions at the end of the cycle. The complete oxygen tension image sequences of each cycle are shown in the left part of Video V1 in the Supporting Information.

Figure 3A presents a sketch of the scaffold and a set of equations for modeling. The sketch shows the scaffold in an imagined bone fracture, where the edge of the fracture provides oxygenation, modeled as a fixed boundary condition and experimentally provided by the reservoir of fluid at the sides of the scaffold in this study. The one-dimensional reaction-diffusion for oxygen tension (eq 1 in Figure 3A) describes the interplay of oxygen diffusion and consumption by the cells inside the scaffold along the *x*-axis.^31^ At high oxygen tensions, the oxygen consumption rate *Q*(*x*,*t*) can be assumed to be invariant over short times, but at low tensions the consumption becomes tension-dependent and can be assumed to follow Michaelis–Menten kinetics,^32^ as described in eq 2. At steady state, the time derivative in eq 1 approaches zero, and the oxygen tension along a line parallel to the scaffold face and crossing the scaffold center (i.e., along *x*) should follow roughly the parabolic equations^33^ eqs 3a and 3b, expressed in dimensionless and dimensional form, respectively.

**Figure 3:**
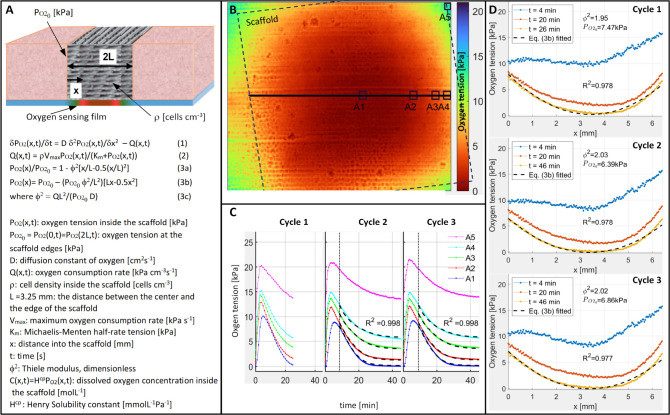
(A) A sketch of the scaffold and equations describing the oxygen tension and concentration inside the scaffold. Here, eq 1 is a one-dimensional diffusion equation with a reaction term *Q*(*x*,*t*); eq 2 presents Michaelis–Menten kinetics for *Q*(*x*,*t*), and eqs 3a and 3b present a stationary solution for eq 1 in nondimensional and dimensional forms, respectively. (B) The oxygen image at the end of Cycle 3 showing the position of the scaffold, the line-profile used in stationary data analysis, and the small regions A1–A5 used in time-dependent data-analysis. (C) The measured oxygen partial pressure values averaged over the areas A1–A5 during the three measurement cycles. The data are shown as connected dots and the fits with a dashed black line. (D) The oxygen concentration along the line-profile shown in (B) at three time points. The dots (·) correspond to data and the dashed black lines represent eq 3b with the best-fit parameter values. The calculated *R* ^2^ values represent the coefficient of determination. The best-fit parameter values are collected in Table 1.

Figure 3B shows the oxygen tension image at the end of Cycle 3 (*t* = 46 min). A dashed black line shows the approximative edges of the scaffold, and a solid black line indicates the centerline *y* = 72, where the line-profile is taken to compare with eq 3b. It is noteworthy that the scaffold is angled at approximately 10° with respect to the camera, but the resulting distortion in spatial coordinates is small (∼1.5%) and was omitted in further analysis. Figure 3D shows the line-profile data at three time points after flushing: 1 min after the flushing ends (*t* = 4 min), when *P*_O2_ values peak, 17 min after the flushing ends (*t* = 20 min), and finally close to the steady state (the last time stamp, *t* = 26 min for Cycle 1 and *t* = 46 min for Cycles 2 and 3). Here, the line-profiles start and end approximately at the edges of the scaffold, and the plots represent the averages of 11 line-profiles between *y* = 67 and *y* = 77 (an average of *y* = 72 with height of 10 pixels or 0.36 mm). The center of the scaffold depletes more than the edges, reaching strongly hypoxic conditions close to *P*_O2_ = 0.0 kPa. Additionally, the steady state data fits well to eq 3b (*R* ^2^ > 0.97), and the data are reproducible from cycle to cycle, with shorter 26 min Cycle 1 giving ϕ^2^ = 1.95, and longer 46 min Cycles 2 and 3 giving ϕ^2^ = 2.03 and ϕ^2^ = 2.02, respectively. Finally, while Figure 3D shows only line profiles at *y* = 72, the profiles between *y* = 62 and *y* = 102 would produce very similar results, as the supplementary video of the multiple developing line-profiles shows (Video V1, the right part).

We next studied the oxygen dynamics at different locations. Figure 3B shows five 0.22 × 0.22 mm square regions of interest labeled A1–A5. Here, the regions A1–A4 are located along the line-profile beneath the scaffold, and A5 is in the upper right corner at the edge of the scaffold where the flushing is most efficient. Figure 3C shows the average oxygen tension in each region over time for each flushing and waiting cycle. As before, the starting point *t* = 0 represents the start of the flushing step in each cycle. During the 3 min flushing, oxygen tension increased in all regions. The maximum value for the corner region at the edge of the scaffold (A5) reached values close to *P*_O2_ ≈ 21 kPa of dry laboratory conditions, while the region in the scaffold center (A1) peaks lower at *P*_O2_ ≈ 9.5 kPa. After flushing ceases, the oxygen levels fall again as metabolic activity of the yeast depletes the available oxygen. The center region (A1) experiences a brief delay of approximately 2 min where the oxygen tension appears to rise slowly and peak even after the flushing has ceased and oxygen tension is falling elsewhere in the scaffold. The most likely reason for this is diffusion from a more oxygenated neighboring region. Assuming oxygen diffusion is at most the diffusion constant in free water (∼2 × 10^–5^ cm^2^/s),^34^ 1-D oxygen diffusion distance in 2 min would be ∼700 μm. This is likely an overestimate since scaffold porosity decreases effective diffusion constants, especially if pores are partially clogged by cells. Since no high concentration oxygen reservoir is evident within 700 μm of A1 in the 2D oxygen images, it is likely that this represents a reduced vertical diffusion to the bottom of the scaffold at this location. In any case, the close agreement in depletion time across all regions (within 2 min) shows reasonably fast and uniform response throughout the scaffold.

After the flushing ceases (and up to a 2 min delay), the oxygen tension decreases in all regions due to the cellular metabolic activity. In the center region (A1), oxygen depletes faster than compensatory diffusion from all neighboring regions, and the oxygen tension approaches zero (hypoxic/necrotic center). The decrease rate appears constant until about 2 kPa (at about 25 min). This is consistent with a Michaelis–Menten depletion mechanism commonly used to describe metabolic activity. At other points closer to the scaffold edge (A2–A4), the oxygen concentration does not become as hypoxic as the center and reaches the steady state in around 30 min. Overall, the response is somewhat slower than in the center (where oxygen depletion is limited by oxygen-dependent metabolic activity), and decay curves are approximately exponential with a time constant of approximately 30 min (Figure 3C).

The dashed black curves represent a 1-D diffusion/Michaelis–Menten model, i.e. a system described by eqs 1 and 2, fitted to the total data set of all four regions inside the scaffold (regions A1–A4). Here we estimated initial values *P*_O2_(*x*,0) from the data of each region at 4 min after *P*_O2_ peaking, and applied fixed boundary conditions *P*_Ο2_ = *P*_O2_(0,*t*) = *P*_O2_(2L,*t*) = 12.2 kPa (the mean of the initial values of the region A4) and used three free variables: the effective diffusion constant of oxygen (*D*), the product of Michaelis–Menten maximum consumption rate and average cell density inside the scaffold (ρ*V*_max_), and Michaelis–Menten half-rate oxygen tension (*K*_m_). While it is evident from Figure 3D and the supplementary video (Video V1, right part) that the true boundary in our experiment is not fixed but changes in time, a fixed boundary condition with a value close to the real concentration values at the scaffold edges at the end of the flushing phase nevertheless provides a sound straightforward starting point for the dynamical modeling. The best-fit parameter values for Cycles 2 and 3 are shown in Table 1. Overall, the model agrees well with the decaying oxygen tension data (*R* ^2^ > 0.99), suggesting that the settling oxygen concentrations after flushing inside a cell-containing scaffold can be accurately described with the 1-D diffusion/Michaelis–Menten model. Interestingly, the best-fit diffusion constant values (*D* = 1.78 × 10^–5^ cm^2^/s for Cycle 2 and *D* = 1.76 × 10^–5^ cm^2^/s for Cycle 3 are approximately 10% lower than the oxygen diffusion constant in free water. The relatively close agreement suggests that the reaction-diffusion model can explain the experiment. At the same time, exact accord is not expected, as there are effects that would both increase the measured constant and decrease compared to 1D diffusion: On the one hand, lower diffusion is expected from the ∼43% porosity in the scaffold which would reduce diffusive flow, especially in clogged regions; on the other hand, diffusion in 2D or 3D would speed oxygen transport compared to 1D and is evident in the shape of the depletion region (the corners of the scaffold are more oxygenated than the edges, implying some *y*-direction diffusion is occurring); additionally, any convection would increase oxygen transport. The Michaelis–Menten half-rate parameter has best-fit values of *K*_m_ = 2.83 kPa and *K*_m_ = 2.95, respectively, which confirms our observations above about the shape of the oxygen tension decrease for the central region A1. Finally, the product of the cell density and maximum depletion rate ρ*V*_max_ has the best-fit value of 0.016 kPa s^–1^ cm^–3^ for both cycles. Solving eq 3c for *Q* yields *Q* = *DP*_Ο2_0__ϕ^2^/*L* ^2^, and substituting *D* = 1.8 × 10^–5^ cm^2^/s, *P*_Ο2_0__ = 12 kPa, ϕ^2^ = 2, and *L* = 0.325 cm results in a value of *Q* ≈ 0.004 kPa s^–1^ cm^–3^. Here, the average oxygen consumption rate *Q*, estimated from the end-point data and the stationary eq 3 alone, is approximately 25% of the estimated maximum oxygen consumption rate ρ*V*_max_ after the flushing.

**Table 1:** Best-Fit Parameter Values for the Three Measurement Cycles

parameter	eq	Cycle 1	Cycle 2	Cycle 3
*D* [cm^2^ s^–1^]	(1), (2)		1.78 × 10^–5^	1.76 × 10^–5^
ρ*V*_max_ [kPa s^–1^ cm^–3^]	(1), (2)		0.016	0.016
*K*_m_ [kPa]	(1), (2)		2.83	2.95
ϕ^2^	(3b)	1.95	2.03	2.02

In terms of the cellular activity, a wide range of Michaelis–Menten parameters are reported for similar systems, and some reports indicate that they depend on cellular concentration.^35^ Although we used robust yeast cells with a nutrient concentration to remove nutrient depletion and waste accumulation variables, they could also contribute. Nonetheless, it is clear from the oxygen image sequences that oxygen diffuses from the edges leading to hypoxic regions <5 kPa within 1–2 mm of the scaffold edge. In the center, the rate of depletion slows as metabolic activity decreases in hypoxic regions. Additionally, perfusion increases oxygen tension throughout the scaffold including the necrotic core. This is an important finding, as it suggests that approaches including external perfusion could be useful at oxygenating implanted scaffolds.

In conclusion, this work has demonstrated the power of ratiometric oxygen imaging beneath robocast bone scaffolds at a high spatial and temporal resolution. We observe depletion due to cellular activity and restoration from perfusion with high cycle-to-cycle reproducibility. Similar studies on 2D oxygen imaging of bacterial biofilms under varying flow conditions have been reported, albeit with much thinner cell mass and luminescence lifetime imaging microscopy.^36^

Future work involves careful study of bone cell culture and infection models to understand how changes in perfusion affect cell growth and pathology. In these *in vitro* studies, distributed optical O_2_ sensor nanoparticles^37^ combined with 3D-microscopies could prove useful, if the scaffold and the tissue are transparent enough. Such sensor particles would allow not only for imaging of the 3D oxygen distribution but also for discarding the oxygen sensing plate, which acts as a wall and blocks both the diffusive and advective flow vertically through the scaffold, while increasing viscous drag. Furthermore, the optical sensing particles can simultaneously provide means to image perfusion induced flow fields through particle image velocimetry (PIV), a technique recently introduced by Ahmerkamp and coauthors.^38^ Finally, we will also expand the system to oxygen sensing X-ray luminescence imaging for animal studies.

## Supplementary Material







## Notes

Images of the red (a) and green (b) channels, and the corresponding ratiometric image (c) taken in the beginning of Cycle 2 (TIF)
Calibration curves (TIF)
Video showing the complete oxygen image sequences (the left part) and developing line profiles (the right part) of all cycles (AVI)
