# Supplementary figures and images for: Imaging Oxygen Concentrations in Bone Scaffolds during Cellular Activity and Fluid Perfusion
Source: ACS Biomater Sci Eng. 2025 Jun 4;11(7):4050–6. doi: 10.1021/acsbiomaterials.4c01845 (PMC12264768; doi:10.1021/acsbiomaterials.4c01845)

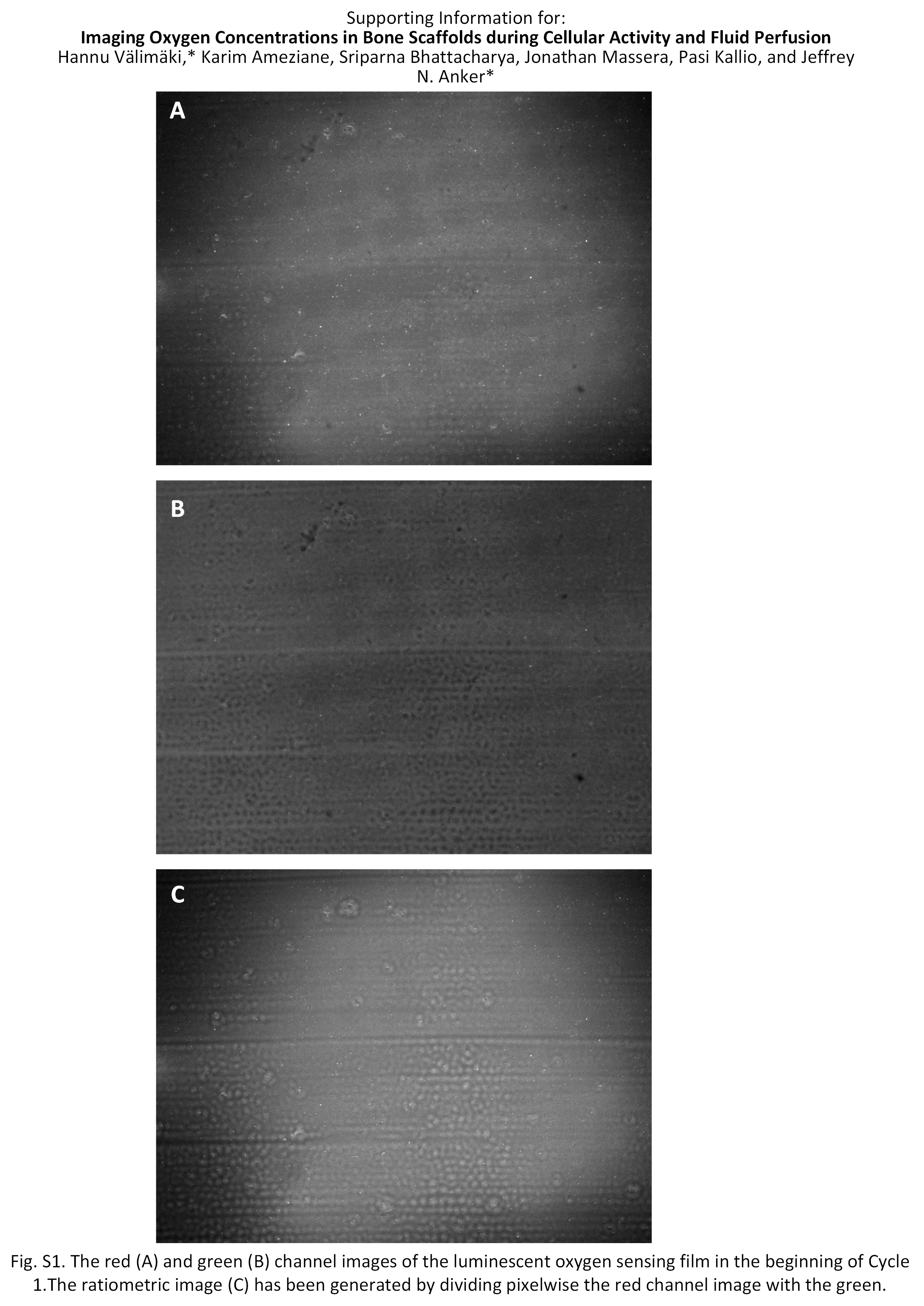

Supplement: Supplementary file 1 [file ab4c01845_si_001.tif]

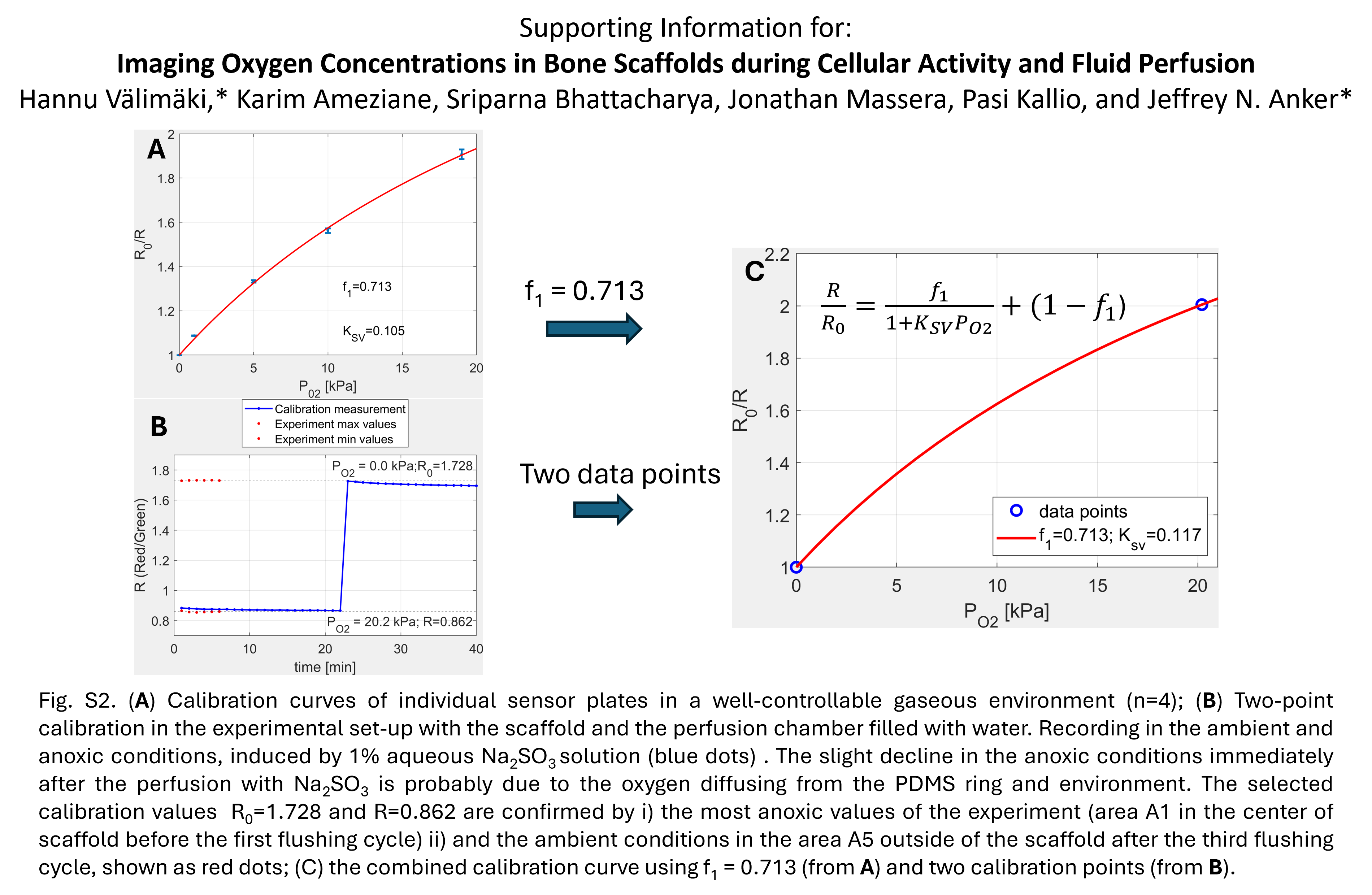

Supplement: Supplementary file 2 [file ab4c01845_si_002.tif]
